# Neutrophils drive alveolar macrophage IL-1β release during respiratory viral infection

**DOI:** 10.1136/thoraxjnl-2017-210010

**Published:** 2017-10-27

**Authors:** Teresa Peiró, Dhiren F Patel, Samia Akthar, Lisa G Gregory, Chloe J Pyle, James A Harker, Mark A Birrell, Clare M Lloyd, Robert J Snelgrove

**Affiliations:** 1 Inflammation, Repair and Development Section, National Heart and Lung Institute, Imperial College London, London, UK; 2 Respiratory Pharmacology, Airway Disease Section, National Heart and Lung Institute, Imperial College London, London, UK

**Keywords:** innate immunity, macrophage biology, neutrophil biology, respiratory infection, viral infection

## Abstract

**Background:**

Alveolar macrophages are sentinels of the airways that must exhibit immune restraint to innocuous antigens but elicit a robust inflammatory response to pathogenic threats. How distinction between these dichotomous functions is controlled is poorly defined.

Neutrophils are the first responders to infection, and we hypothesised that they may free alveolar macrophages from their hyporesponsive state, promoting their activation. Activation of the inflammasome and interleukin (IL)-1β release is a key early inflammatory event that must be tightly regulated. Thus, the role of neutrophils in defining inflammasome activation in the alveolar macrophage was assessed.

**Methods:**

Mice were infected with the X31 strain of influenza virus and the role of neutrophils in alveolar macrophage activation established through administration of a neutrophil-depleting (1A8) antibody.

**Results:**

Influenza elicited a robust IL-1β release that correlated (r=0.6849; p<0.001) with neutrophil infiltrate and was ablated by neutrophil depletion. Alveolar macrophages were shown to be the prominent source of IL-1β during influenza infection, and virus triggered the expression of Nod-like receptor protein 3 (NLRP3) inflammasome and pro-IL-1β in these cells. However, subsequent activation of the inflammasome complex and release of mature IL-1β from alveolar macrophages were critically dependent on the provision of a secondary signal, in the form of antimicrobial peptide mCRAMP, from infiltrating neutrophils.

**Conclusions:**

Neutrophils are critical for the activation of the NLRP3 inflammasome in alveolar macrophages during respiratory viral infection. Accordingly, we rationalise that neutrophils are recruited to the lung to confront a viable pathogenic threat and subsequently commit alveolar macrophages to a pro-inflammatory phenotype to combat infection.

**Key messages**

**What is the key question?**

Do neutrophils, as first responders, release alveolar macrophages from their hyporesponsive state and enable them to respond more robustly upon influenza infection?

**What is the bottom line?**

IL-1β release during influenza infection is critically dependent on neutrophil-driven activation of the NLRP3 inflammasome in alveolar macrophages and is mediated through the neutrophil antimicrobial peptide mCRAMP.

**Why read on?**

This neutrophil-alveolar macrophage crosstalk represents a novel mechanism by which neutrophils, recruited to confront a viable pathogenic threat, play a decisive role in liberating alveolar macrophages from their hyporesponsive state and amplifying inflammation.

## Introduction

Alveolar macrophages represent the predominant immune cell in homeostatic airways and must exhibit immune restraint to innocuous antigens to limit host tissue damage and preserve pulmonary function. Alveolar macrophages reside in close proximity to the airway epithelium, which supports this hyporesponsive state through direct cell contact or release of soluble mediators.^1^ In the face of a pathogenic threat, however, alveolar macrophages must overcome this hyporesponsive state to elicit a robust inflammatory response to protect the host.^2^ How distinction between these dichotomous functions is controlled remains poorly defined.

Neutrophils are the first responders to infection, being readily recruited to the lung to kill invading micro-organisms through an array of strategies.^3 4^ However, due to the indiscriminate nature of their products, an overexuberant neutrophilic response can also cause bystander tissue damage. Neutrophils are a prominent early feature of influenza viral infection,^5^ but their role remains controversial, with both protective^6^ and pathological^7^ contributions ascribed. It is now clear that neutrophils exhibit remarkable plasticity and are capable of shaping diverse aspects of the inflammatory response via interactions with a multitude of cells.^8 9^ Among these interactions, neutrophils have been described to modulate macrophage activity,^10^ raising the possibility that neutrophils may be involved in the regulation of alveolar macrophage function.

The inflammasome-mediated production of IL-1β is a key effector mechanism in host defence. Additionally, recent studies have highlighted associations between inflammasome activation and IL-1β production in chronic lung diseases such as COPD and asthma (for excellent brief review, see Kim *et al*^11^). Furthermore, inflammasome-dependent IL-1β release has recently been shown to be important in driving neutrophilic inflammation and airway hyper-responsiveness in infection-associated severe steroid-resistant asthma.^12^ Inflammasomes are multiprotein complexes required for the processing and release of mature IL-1β and IL-18 through the activation of caspase-1.^13^ Among the inflammasome family, the well-characterised Nod-like receptor protein 3 (NLRP3) inflammasome has three subunits (the sensor protein NLRP3, the adaptor protein apoptosis-associated speck-like protein (ASC) and the caspase-1 zymogen) and requires two distinct signals for its activation. Signal 1 induces the expression of the inflammasome components and IL-1β and IL-18 pro-forms, while signal 2 leads to inflammasome assembly, caspase-1 auto-cleavage and mature IL-1β and IL-18 release.^14^ The NLRP3 inflammasome-mediated IL-1β release is a prominent feature of macrophages.^15–17^ However, neutrophils have also been reported to release IL-1β through the NLRP3 inflammasome pathway.^18 19^

The NLRP3 inflammasome is central to influenza-induced IL-1β production,^20–22^ with macrophages anticipated to be the prominent source of IL-1β.^15 20 23–25^ An abundance of literature has described the fundamental role of IL-1β in host defence against influenza^20 21 25 26^; however, excess IL-1β could play a pathological role.^27^ The influenza virus provides signal 1 that promotes expression of inflammasome components, via the activation of toll-like receptor 7 (TLR7).^25^ However, the source of signal 2 during influenza infection is less clear, although virus encoded M2 and PB1-F2 proteins have been suggested to be capable of triggering inflammasome activation.^14 28^

We hypothesised that neutrophils may release alveolar macrophages from their hyporesponsive state and enable them to respond more robustly upon influenza infection. In this paper, we demonstrate that IL-1β release during influenza infection is critically dependent on neutrophil-driven activation of the NLRP3 inflammasome in alveolar macrophages and is mediated through the neutrophil antimicrobial peptide mCRAMP.

## Methods

### Murine *in vivo* infection models

All animal studies conformed to ARRIVE guidelines. Female BALB/c mice, 6–8 weeks old, were purchased from Harlan Olac (Oxon, UK). Caspase-1 knock-out mice and littermate controls (C57BL/6 background) were bred in-house. All mice were kept in specific pathogen-free conditions and provided autoclaved food, water and bedding. This study was carried out in accordance with the recommendations in the Guide for the Use of Laboratory Animals of Imperial College London. All animal procedures and care conformed strictly to the UK Home Office Guidelines under the Animals (Scientific Procedures) Act 1986, and the protocols were approved by the Home Office of Great Britain. Since all experiments were conducted in female mice, we are unable to unequivocally state whether the observed results will be conserved in males. Consequently, the inability to investigate gender differences is a limitation of this study. Each mouse represented an individual unit of analysis for each dataset. For each experiment, there were five mice per group. The primary outcome and most variable readout of the study was IL-1β levels. Based on analysis of a large historical dataset, this readout was normally distributed with a relative SD of approximately 23%. With a level of significance of 0.05, we would be able to detect a difference of 50% between influenza-treated groups with a power of 0.85. The number of groups within each experiment and the number of individual replicates are specifically defined within the figure legends detailing the respective studies. Balb/c mice were anaesthetised with isoflurane and infected intranasally (i.n.) with influenza virus strain X31 (1×10^5^ pfu in 50 µl of phosphate-buffered saline (PBS)), respiratory syncytial virus (RSV; 8×10^5^ pfu in 100 µl of PBS), *Streptococcus pneumoniae* (SP) (1×10^6^ cfu in 50 µl of PBS), *Haemophilus influenzae* b (Hib) (1×10^7^ cfu in 50 µl of PBS) or lipopolysaccharide (LPS; 10 µg in 50 µl of PBS) and culled at times denoted in the text. Details of pathogens, culture and pathogen enumeration and influenza-specific plaque assay are described in the online supplementary methods. In neutrophil depletion experiments, mice received intraperitoneal (i.p.) administration of 100 µg of anti-Ly6G (1A8, Bio X Cell, West Lebanon, NH, USA) or control rat IgG antibody (2A3, Bio X Cell) the day preceding infection. In other experiments, mice were treated with allopurinol (Sigma-Aldrich; 25 mg/kg; i.p.) or suramin (Sigma-Aldrich; 2 mM in 50 µl PBS; i.n.) followed by infection with influenza virus i.n. or PBS 1 hour after treatment. The allopurinol group received another dose 8 hours post infection. Details on cell recovery, isolation and phenotyping by flow cytometry are described in the online supplementary methods.

10.1136/thoraxjnl-2017-210010.supp1 Supplementary file 1

### IL-1β immunofluorescence staining

Paraffin sections of lung tissue were stained with goat anti-IL-1β antibody (AF-401-NA; R&D Systems, Oxon, UK) followed by secondary donkey anti-goat IgG Alexa Fluor 488 conjugate. Additional details are provided in the online Supplementary Methods.

### Alveolar macrophage and bone marrow neutrophil *in vitro* stimulations

Isolated alveolar macrophages and neutrophils were stimulated to assess inflammasome activation and IL-1β release. Cells were primed for 4 hours with 1 µg/mL TLR7 agonist Imiquimod R837 (Invitrogen, France; to mimic signal one required for inflammasome induction) or vehicle. Cells were subsequently cultured with 1 mM ATP (Sigma-Aldrich) or 20 µM mCRAMP (Innovagen, Sweden; to mimic signal 2 required for inflammasome activation) or vehicle. Additional details are provided in the online Supplementary Methods.

### Mediator analysis

The levels of cytokines (IL-1β, tumour necrosis factor-α (TNF-α), IL-6), damage marker lactate dehydrogenase and damage-associated molecular patterns (DAMPs) (ATP and uric acid) were measured in bronchoalveolar lavage fluid (BALF), lung homogenates or cell-free culture supernatants as described in the online Supplementary Methods.

### RNA extraction and real-time PCR

Total RNA was extracted from lung tissue or isolated cells using Qiagen RNeasy kits and reverse transcribed into cDNA using a High Capacity cDNA Reverse Transcription kit (Life Technologies, Paisley, UK). Real-time PCR reactions were performed using fast-qPCR mastermix (Life Technologies) on a Viaa-7 instrument (Life Technologies) with TaqMan primer sets for IL-1β, NLRP3, caspase-1, ASC, hypoxanthine phosphoribosyl transferase (HPRT) and GAPDH transcript levels as described in the online Supplementary Methods.

### Western blot

IL-1β expression was assessed in cultured supernatants using polyclonal goat anti-IL-1β antibody (AF-401-NA; R&D Systems). mCRAMP expression was assessed in BALF using polyclonal rabbit anti-mCRAMP antibody (PA-CRPL; Innovagen). Caspase-1 expression was assessed in lung cytoplasmic fractions using an anti-caspase-1 (p20) antibody. This was followed by incubation with appropriate secondary horseradish peroxidase-linked antibodies. Additional details are provided in the online Supplementary Methods.

### Statistics

Data were assessed for normal distribution using the Shapiro-Wilk normality test and found to be non-normal. For comparison between two groups, statistical significance was calculated with a non-parametric Mann-Whitney test (two sided). Group comparisons were performed using a non-parametric analysis of variance test (Kruskal-Wallis), followed by a Dunn’s post-test for multiple comparisons between groups. Details of the specific statistical test used for each experiment are described in respective figure legends. Results are depicted as median±IQR unless stated otherwise. Correlations were calculated using a Spearman’s rank correlation coefficient for non-parametric data. All reported p values are two sided, and p<0.05 was considered significant and is referred to as such in the text. p Values are depicted in the graphs. Graph generation and statistical analyses were performed using GraphPad Prism software (GraphPad Software).

## Results

### Influenza elicits an early release of IL-1β that is critically dependent on neutrophil recruitment

**Figure 1:**
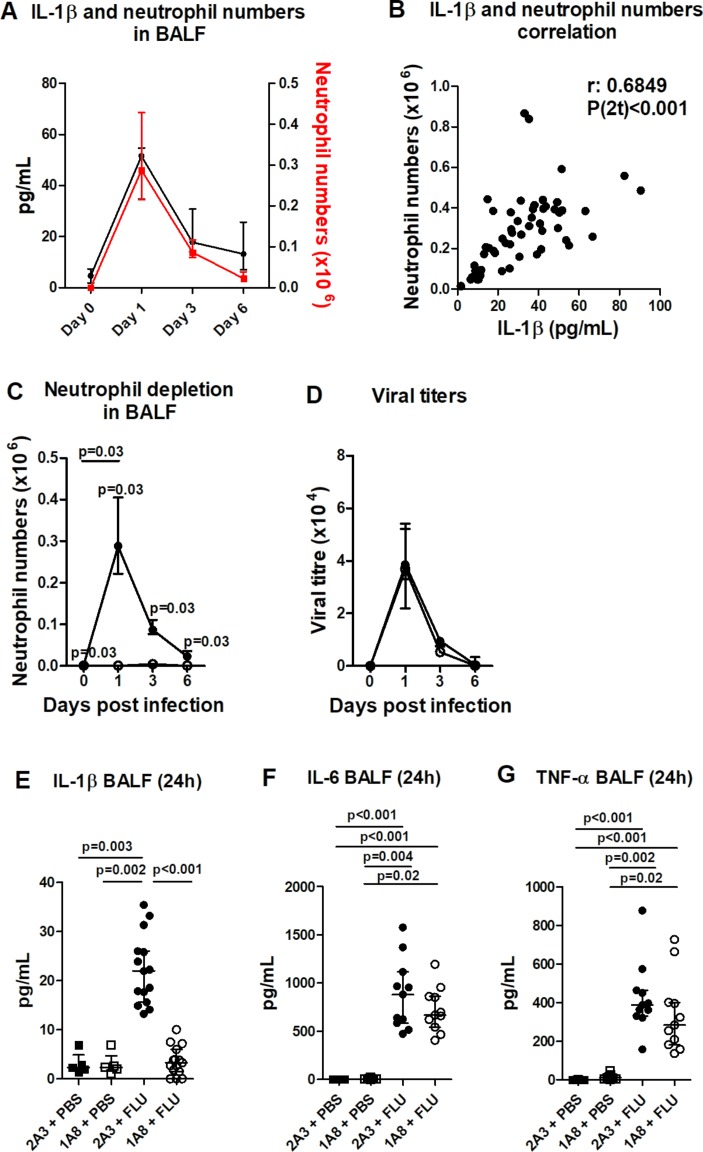
Influenza infection elicits a robust IL-1β release that is dependent on neutrophil recruitment. Mice were inoculated with influenza intranasally (i.n.) and culled at days 1, 3 and 6 post infection. (A) Total IL-1β in the bronchoalveolar lavage fluid (BALF) and the number of neutrophils recruited into the airways were determined by ELISA and flow cytometry, respectively. (B) Correlation between IL-1β and neutrophil infiltrate in BALF at 24 hours post infection. Mice were administered neutrophil-depleting (1A8) (open symbols) or control (2A3) antibody (filled symbols) intraperitoneally (i.p.) and inoculated with influenza X31 (1×10^5^ pfu in 50 µl PBS) or PBS i.n. and culled at days 1, 3 and 6 post infection. (C) BALF neutrophil numbers were determined by flow cytometry. (D) Plaque assay of lung viral titres in control and neutrophil-depleted mice on days 1, 3 and 6 after infection with influenza. The concentration of (E) IL-1β, (F) IL-6 and (G) TNF-α in the BALF were determined by ELISA. Figures represent (A) data from one experiment with five mice per group (representative of two independent experiments); (B) Spearman correlation from 11 experiments with five mice per group; (C, D) data from one experiment with five mice per group and representative of two independent experiments; (E) data combined from three experiments (representative of eight independent experiments); (F, G) data combined from two experiments. Results are depicted as median±IQR. p Values were calculated using Mann-Whitney statistical test (A, C and D) or Kruskal-Wallis with Dunn’s post-test (E–G).

In a murine model of X31 influenza infection, neutrophils peak in the airways at 24 hours post infection (figure 1A), coinciding with peak viral titres (24–48 hours) but preceding peak illness and inflammation (day 6).^29^ BALF IL-1β levels longitudinally mapped with neutrophil infiltrate during influenza infection (figure 1A), and there was a significant correlation between IL-1β levels and neutrophil numbers at 24 hours post infection (figure 1B). To investigate the role of neutrophils in mediating IL-1β release, mice were administered neutrophil-depleting (1A8) or control (2A3) antibody prior to influenza inoculation. Administration of 1A8 completely ablated neutrophils in BALF at 24 hours post infection (figure 1C) without compromising viral titres (figure 1D) and resulted in an ameliorated illness and inflammation by day 6 (see online supplementary figure 1). Strikingly, BALF IL-1β levels in influenza-infected mice at 24 hours post infection were reduced to control levels following neutrophil depletion (figure 1E). This neutrophil dependence for IL-1β release was not seen with other cytokines, with BALF IL-6 and TNF-α levels comparable between influenza-infected control and neutrophil-depleted mice (figure 1F, G). Thus, these data would point to a specific inflammasome phenotype with neutrophil depletion.

### Viral-specific dependence on neutrophils for IL-1β release

**Figure 2:**
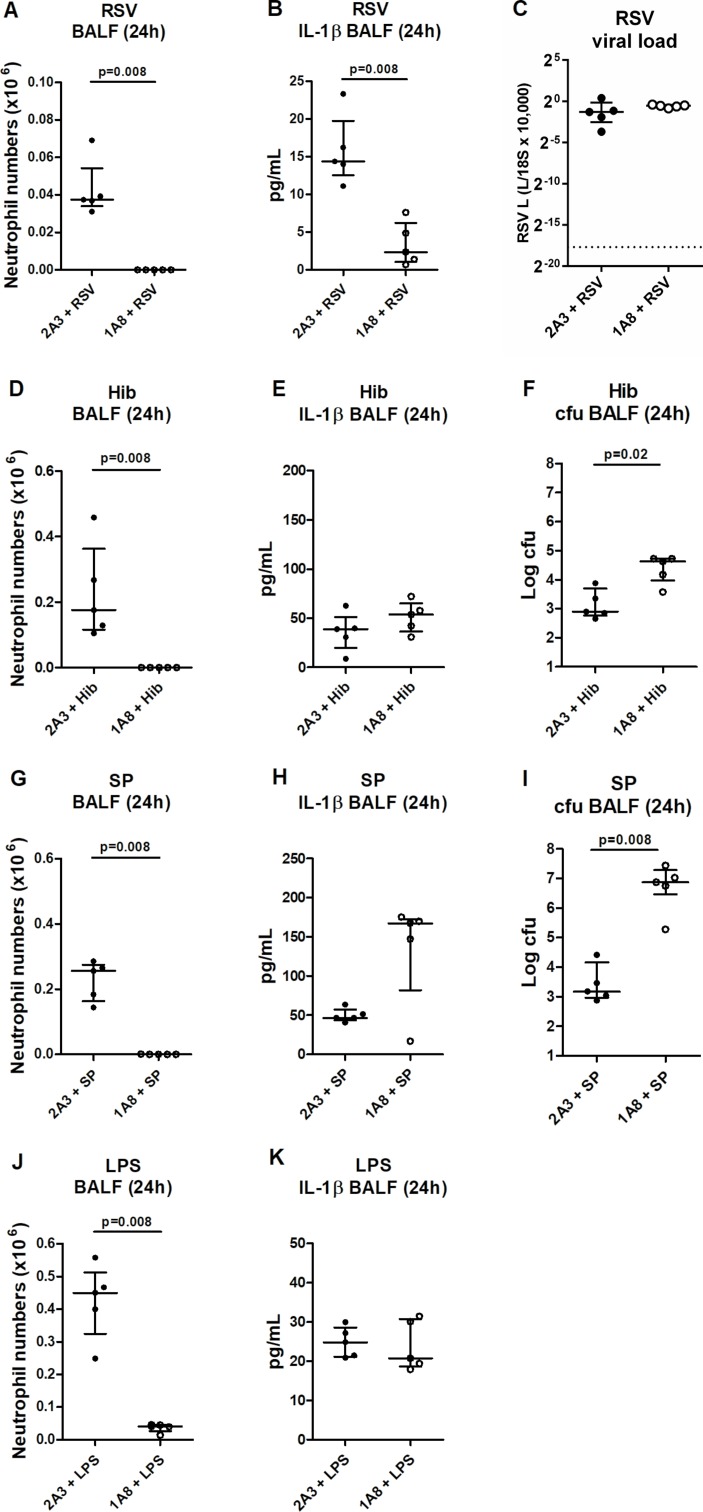
Respiratory virus triggers neutrophil-dependent IL-1β release. Mice were administered neutrophil-depleting (1A8) or control (2A3) antibody intraperitoneally (i.p.) and inoculated intranasally (i.n.) with (A–C) respiratory syncytial virus, (D–F) *Haemophilus influenzae b* (Hib), (G–I) *Streptococcus pneumoniae* (SP) or (J, K) lipopolysaccharide and culled 24 hours later. (A, D, G, J) Bronchoalveolar lavage fluid (BALF) neutrophil numbers were determined by flow cytometry. (B, E, H, K) Total IL-1β in the BALF was determined by ELISA. (C) Viral titres were determined by real-time PCR of lung tissue. (F, I) Bacterial burden (cfu) was determined by performing serial dilutions of BALF on Columbia blood agar plates (SP) or brain–heart infusion agar (Hib). Figures represent (A–K) data from five mice per group. Results depicted as median±IQR. p Values were calculated using Mann-Whitney statistical test.

To infer whether this absolute requirement of neutrophil recruitment for IL-1β release was influenza specific, we assessed the consequence of neutrophil depletion in other respiratory infectious models. Neutrophil depletion also ablated BALF IL-1β at 24 hours post RSV infection (figure 2A, B), without compromising viral titres (figure 2C). In contrast, no reduction in BALF IL-1β was seen in neutrophil-depleted mice treated with bacteria Hib (figure 2D–F) or SP (figure 2G–I), although this could be rationalised by the compromised bacterial clearance in these models (figure 2F, I, respectively). To negate the confounder of altered pathogen clearance, the effect of neutrophil depletion in an LPS model was assessed, but again, BALF IL-1β levels remained unaltered following neutrophil depletion (figure 2J, K). Collectively, these data indicate that some respiratory viral infections elicit a robust release of IL-1β that is critically dependent on neutrophil recruitment. However, this pathway for inflammasome activation is seemingly not conserved for all infectious insults.

### Alveolar macrophages are the prominent source of IL-1β during influenza infection

**Figure 3:**
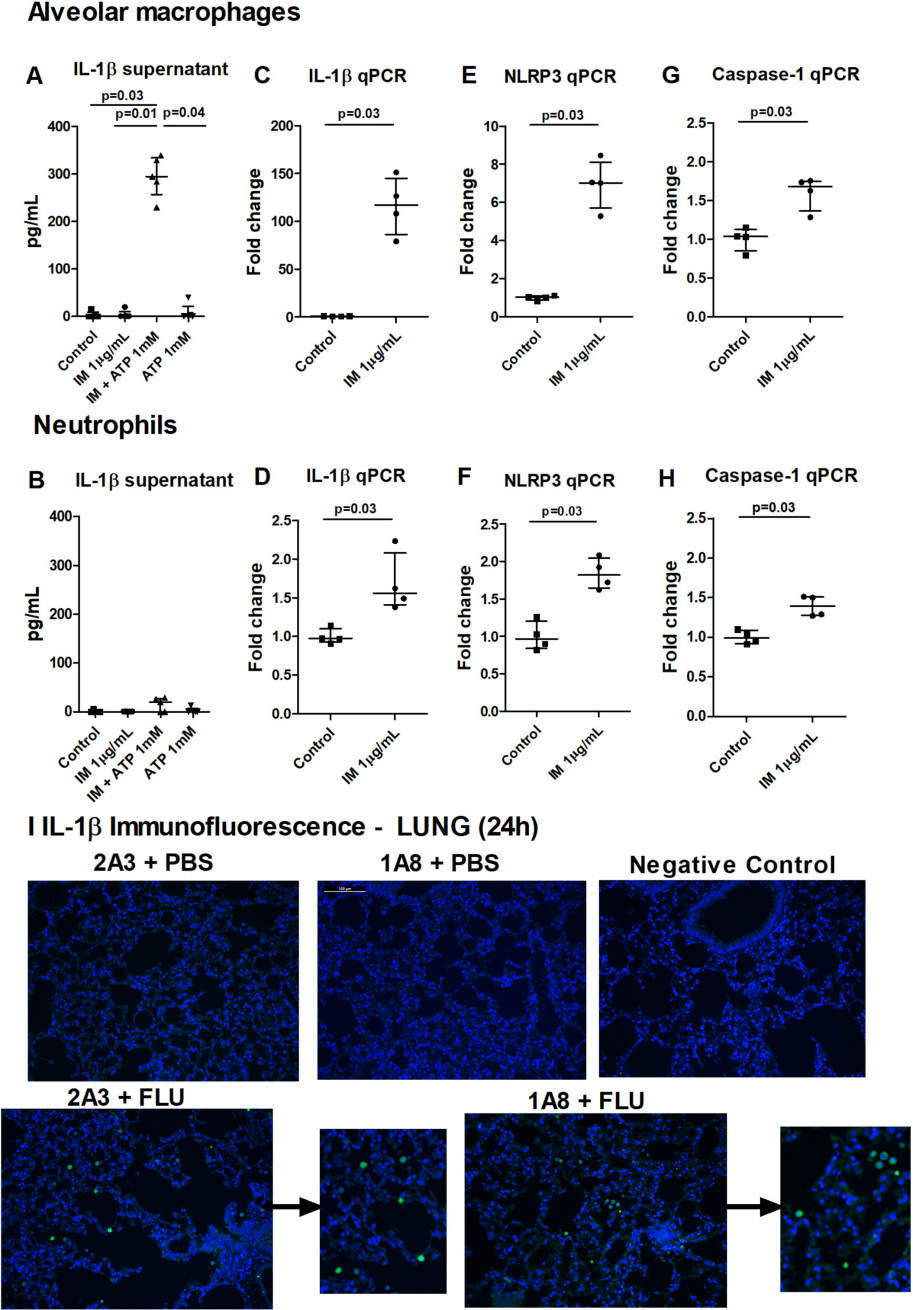
Alveolar macrophages are the source of Nod-like receptor protein 3 (NLRP3) and caspase-1-dependent IL-1β during influenza infection. Alveolar macrophages (AM) (A) or bone marrow (BM) neutrophils (B) were primed with TLR7 agonist imiquimod (IM) for 4 hours followed by stimulation with ATP for 1 hour and ensuing IL-1β release into supernatant assessed by ELISA. Alveolar macrophages (C, E, G) or BM neutrophils (D, F, H) were stimulated with IM for 4 hours, and levels of IL-1β, NLRP3 and caspase-1 messenger RNA assessed by real-time PCR; depicted as fold change relative to HPRT. Mice were administered neutrophil-depleting (1A8) or control (2A3) antibody intraperitoneally and inoculated with influenza or phosphate-buffered saline intranasally and culled 24 hours later. (I) IL-1β expression in alveolar macrophages in paraffin sections of lung tissue from mice treated with influenza. Original magnification ×20; scale 100 µm. Data presented from one experiment with (A, B) n=5 per group and representative of two experiments or (C–H) n=4 per group. Results are depicted as median±IQR. p Values were calculated using Kruskal-Wallis with Dunn’s post-test (A and B) or Mann-Whitney statistical test (C–H).

Lung epithelial cells, endothelial cells and hematopoietic cells have been reported as potential sources of IL-1β. To identify the principle population responsible for IL-1β in our influenza model, we isolated these populations by fluorescence-activated cell sorting (see online supplementary figure 2A). IL-1β expression in the hematopoietic compartment was substantially greater than that observed in epithelial cells or endothelial cells (see online supplementary figure 2B). Given these findings, an absence of IL-1β in neutrophil-depleted mice following influenza infection would suggest that either neutrophils are the sole source of this cytokine or that they indirectly mediate IL-1β expression and/or release in other hematopoietic populations (most likely alveolar macrophages, given previous literature).^15 20 23–25^ We subsequently assessed the relative capacity of these candidate cell types to produce IL-1β. Alveolar macrophages or bone marrow (BM) neutrophils were therefore treated with the TLR7 agonist imiquimod (to mimic influenza in providing the first signal),^25^ followed by incubation with ATP (a common DAMP that provides the second signal). Alveolar macrophages were significantly more potent than neutrophils at producing IL-1β on a per cell basis (figure 3A, B), which was rationalised by a substantially greater induction of IL-1β and NLRP3 messenger RNA (mRNA) by imiquimod in alveolar macrophages (figure 3C, E, G) relative to neutrophils (figure 3D, F, H). As anticipated, alveolar macrophage IL-1β production was reduced by genetic deletion of caspase-1 or NLRP3 inhibition (see online supplementary figure 3A). Given that comparable numbers of alveolar macrophages and neutrophils are observed in the airways at 24 hours post influenza infection in our model (see online supplementary figure 3B–D), it is extremely likely that alveolar macrophages are the prominent source of IL-1β. Examination of IL-1β stained tissue sections from influenza-infected animals further supported the assertion, with IL-1β staining solely restricted to macrophages of influenza-infected mice (figure 3I). These results corroborate the literature in pointing to macrophages as the main source of IL-1β release during influenza infection.^15 20 23–25^

### Neutrophils provide the second signal that activates the NLRP3 inflammasome in alveolar macrophages during influenza infection

**Figure 4:**
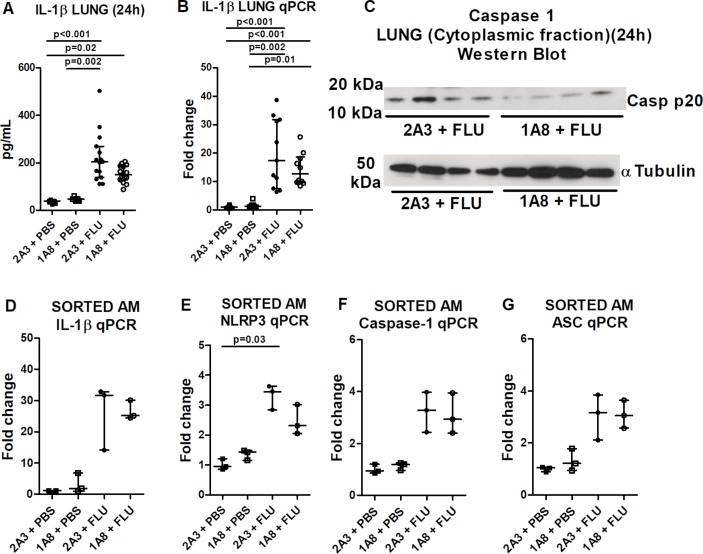
Influenza-induced expression of IL-1β and inflammasome components is maintained following neutrophil depletion. Mice were administered neutrophil-depleting (1A8) or control (2A3) antibody intraperitoneally and inoculated with influenza or PBS intranasally and culled 24 hours later. Total lung IL-1β (A) and IL-1β messenger RNA (mRNA) (B) were determined by ELISA and real-time PCR (fold change relative to HPRT), respectively. (C) Western blot depicting caspase-1 mature p20 or α-tubulin loading control in cytoplasmic fraction of lung homogenates. (D–G) Alveolar macrophages were isolated from bronchoalveolar lavage fluid by magnetic-activatedcell sorting (MACS) and IL-1β, NLRP3, caspase-1 and apoptosis-associated speck-like protein mRNA levels assessed by real-time PCR (fold change relative to GAPDH). The graphs present (A) data combined from three experiments and representative of five independent experiments; (B) data combined from two experiments with at least five mice per group; (D–G) data from two experiments with three to five mice per group. Results are depicted as median±IQR. p Values were calculated using Kruskal-Wallis with Dunn’s post-test.

Subsequently, we questioned why alveolar macrophage IL-1β release was ablated in the absence of neutrophils. Lung tissue IL-1β protein (figure 4A) and mRNA levels (figure 4B) were increased comparably in influenza-infected control and neutrophil-depleted mice. The presence of tissue IL-1β protein and mRNA, despite the complete absence of neutrophils, further validates macrophages as the prominent source of this cytokine and suggests that the first signal which drives the expression of inflammasome components is preserved following neutrophil depletion. Conversely, the second signal driving inflammasome activation and IL-1β release is lacking, with levels of cleaved caspase-1 (generated through inflammasome activation) markedly reduced in the lungs of influenza-infected mice after neutrophil depletion (figure 4C). Furthermore, sorted alveolar macrophages from influenza-infected mice exhibited increased expression of pro-IL-1β (figure 4D) and inflammasome components (figure 4E–G), which was unaltered by neutrophil depletion. Collectively, these data suggest that influenza infection drives the expression of IL-1β/inflammasome components (signal 1) in alveolar macrophages, but the inflammasome is not activated nor IL-1β released (signal 2) in the absence of neutrophils.

### The neutrophil peptide mCRAMP activates the inflammasome in alveolar macrophages to promote IL-1β release

We subsequently attempted to decipher the nature of the signal provided by neutrophils during influenza infection that activated the inflammasome in alveolar macrophages. Several DAMPs, such as ATP or uric acid, are known to act as second signals that activate the NLRP3 inflammasome.^30 31^ It is recognised that neutrophils have the potential to cause significant indiscriminate bystander damage and ensuing release of the aforementioned DAMPs. However, levels of BALF lactate dehydrogenase (a marker for cell death; see online supplementary figure 4A), uric acid (see online supplementary figure 4B) and ATP (see online supplementary figure 4C) in influenza-treated mice were unaltered by neutrophil depletion, and administration of allopurinol (to impair uric acid synthesis) or suramin (a broad spectrum P2 receptor antagonist) failed to diminish IL-1β release (see online supplementary figure 4D).

**Figure 5:**
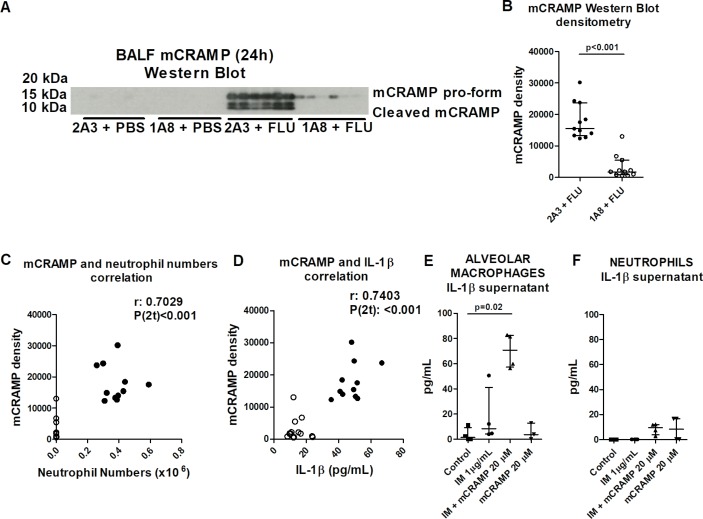
Neutrophil-derived mCRAMP activates the inflammasome in alveolar macrophages during influenza infection. Mice were administered neutrophil-depleting (1A8) or control (2A3) antibody intraperitoneally and inoculated with influenza or PBS intranasally and culled 24 hours later. (A) Amounts of mCRAMP in the BALF were determined by Western blot (representative image from two experiments depicted). (B) Densitometry analysis of mCRAMP expression from Western blot. Correlation between mCRAMP (values from densitometry analysis) and (C) airway neutrophil or (D) BALF IL-1β at 24 hours post infection. Influenza/2A3 group are depicted as closed symbols and influenza/1A8 as open symbols. (E) Alveolar macrophages or (F) bone marrow neutrophils were primed with imiquimod (IM) for 4 hours followed by stimulation with mCRAMP for 2 hours and ensuing IL-1β release into supernatant assessed by ELISA. The graphs present (B) data combined from two experiments with at least five mice per group; (C, D) Spearman correlation from two experiments with at least five mice per group; (E) data combined from two experiments with n=2 per group or (F) one experiment with n=4 per group. Results are depicted as median±IQR. p Values were calculated using Mann-Whitney statistical test (B) or Kruskal-Wallis with Dunn’s post-test (E and F).

The predominantly neutrophil-derived antimicrobial peptide LL-37 and its mouse homologue mCRAMP have been demonstrated to activate the NLRP3 inflammasome in LPS-primed macrophages,^16 32^ and both LL-37 and mCRAMP have previously been purported to play an important antiviral role.^33 34^ In our model, both pro-form and cleaved forms of mCRAMP were present in the BALF of influenza-treated mice 24 hours post infection and were strikingly reduced with neutrophil depletion (figure 5A, B). When analysing all influenza-infected mice (2A3 and 1A8 treated), there was a significant correlation between the levels of BALF mCRAMP and airway neutrophils (figure 5C) or IL-1β (figure 5D). These correlations remained significant when solely analysing the parameters within the influenza/2A3 group (r=0.3859 and p(2 t)=0.03 for mCRAMP vs neutrophil numbers; r=0.4427 and p(2 t)=0.01 for mCRAMP vs IL-1β), supporting the notion that neutrophil mCRAMP may drive IL-1β release from alveolar macrophages. Accordingly, mCRAMP stimulation of imiquimod-primed alveolar macrophages, but not neutrophils, resulted in IL-1β release (figure 5E, F).

## Discussion

Alveolar macrophages are sentinels of the airways that are actively restrained by the localised microenvironment so as to limit activation and prevent unnecessary tissue damage.^1^ However, when confronted with a viable pathogenic threat, alveolar macrophages must elicit a rapid and robust effector programme to protect the host from a foreign genome. Alveolar macrophage inflammasome-mediated production of IL-1β is a critical host defence mechanism, but this pathway must be rigorously controlled to limit ensuing immunopathology. Here, we describe a novel concept whereby neutrophils are readily recruited to the airways during respiratory viral infection and subsequently function to promote alveolar macrophage responsiveness through mCRAMP-mediated activation of the NLRP3 inflammasome. Consequently, neutrophils are crucial in defining the balance between inflammasome-mediated immunity and immunopathology.

The role of neutrophils during influenza infection is unclear, with both protective and pathological roles ascribed,^6 7 35–38^ with disparate findings likely influenced by various factors including the strain and dose of the virus used, the strain of mouse used, and the method and treatment regimen used to deplete neutrophils. In our model, neutrophil depletion did not compromise viral titres but ameliorated illness as a consequence of reduced inflammation and immunopathology. Similarly, while IL-1β has been previously described to have a fundamental role in host defence against influenza,^20 21 25 26^ other studies have demonstrated that an overzealous production of IL-1β may be pathological.^27^ We have not assessed the consequence of specifically neutralising IL-1β during influenza infection in this study. While depletion of neutrophils ablates the IL-1β signal, loss of this cell type is also likely to have a multitude of other effects—some likely beneficial to viral clearance and others likely detrimental. Indeed, while we demonstrate that neutrophils activate the inflammasome in macrophages during influenza infection, other studies have also shown that neutrophils can inhibit macrophage inflammatory responses to viruses.^39^ Regardless, in our physiologically relevant model, influenza elicited an early release of IL-1β that peaked at 24 hours post infection, which was completely dependent on neutrophil infiltrate.

While NLRP3 inflammasome-mediated IL-1β release is a prominent feature of macrophages,^15–17^ neutrophils have also been reported to release IL-1β through the NLRP3 inflammasome pathway.^18 19^ The absence of IL-1β in neutrophil-depleted mice following influenza infection suggested that either neutrophils were the sole source of this cytokine or they indirectly mediate IL-1β expression and/or release in macrophages. An abundance of literature has demonstrated that the NLRP3 inflammasome is central to influenza-induced IL-1β production,^20–22^ with alveolar macrophages deemed to be the prominent source of the cytokine,^15 20 23–25^ and we provide several lines of evidence to support this assertion. First, alveolar macrophages were significantly more potent than neutrophils at producing IL-1β on a per-cell basis, due to a greater induction of IL-1β and NLRP3 mRNA (and both populations were present in comparable numbers in the airways of influenza-infected mice). A caveat of this is that mouse BM neutrophils, while mature and functionally competent,^40^ may not be truly representative of neutrophils present in the airways during influenza infection, and in some severe infections, the numbers of neutrophils may be substantially greater than the numbers of alveolar macrophages. Second, however, we also demonstrate that levels of IL-1β protein in lung tissue of influenza infected mice were unperturbed by neutrophil depletion—levels of the cytokine would be reduced if neutrophils were a prominent source. Finally, immunohistochemical IL-1β staining was solely restricted to macrophages of influenza-infected mice in our model. Collectively, therefore, our results suggest that while alveolar macrophages were the prominent source of IL-1β, in the absence of neutrophils, the macrophages do not release mature cytokine.

The NLRP3 inflammasome requires two distinct signals for its activation. Signal 1 induces the expression of the inflammasome components and IL-1β and IL-18 pro-forms, while signal 2 leads to inflammasome assembly, caspase-1 auto-cleavage and mature IL-1β and IL-18 release.^14^ Since the mRNA levels of IL-1β and inflammasome components were comparable in both the lungs and sorted alveolar macrophages of influenza-infected control and neutrophil-depleted mice, this would support the assertion that provision of first signal is unaltered by absence of neutrophils. Accordingly, the markedly reduced levels of cleaved caspase-1 in the lungs of influenza-infected mice after neutrophil depletion is indicative of the absence of the second signal driving inflammasome activation and IL-1β release. Thus, neutrophils are seemingly critical for the provision of the second signal that activates the NLRP3 inflammasome in alveolar macrophages during influenza infection.

The necessity for an exogenous signal to drive inflammasome activation during influenza infection is at discord to a previous study that suggested the virus itself provides the two signals required for inflammasome expression and activation—with the second signal provided by viral M2 protein.^25^ However, this previous study used BM-derived macrophages in an *in vitro* context and thus may not truly reflect the physiological response in the airways, since alveolar macrophages are a unique population that are extensively regulated by the microenvironment in which they reside. Another study proposed that the influenza PB1-F2 protein can activate the NLRP3 inflammasome.^27^ Although an isogenic X31 influenza lacking PB1-F2 (ΔPB1-F2) elicited a diminished IL-1β release in mice, this virus also provoked a diminished airway neutrophilia, which could contribute to the reduced IL-1β in light of our current findings. This study also demonstrated that PB1-F2 peptide from pathogenic PR8 influenza could drive IL-1β release from BM-derived macrophages that had been primed with LPS, although this required extensive aggregation of the PR8 PB1-F2 peptide that may not occur *in vivo* with X31.

Neutrophils have the potential to cause significant indiscriminate bystander damage that may result in the release of DAMPS, such as ATP or uric acid, which activate the NLRP3 inflammasome.^30 31^ Thus, we postulated that this may be an indirect mechanism by which neutrophils provide the second signal that activates the NLRP3 inflammasome in alveolar macrophages. However, the levels of these DAMPs in the BALF of mice in our influenza model were unaltered by neutrophil depletion, and administration of inhibitors that abrogated these pathways failed to diminish IL-1β release. Subsequently, we questioned whether a neutrophil-derived product could instead directly activate the NLRP3 inflammasome in the alveolar macrophage during influenza infection. Neutrophils have previously been described to activate the inflammasome in macrophages in diseases such as lupus, atherosclerosis and acute lung injury.^16 17 41^ The predominantly neutrophil-derived antimicrobial peptide LL-37 induces IL-1β release from isolated LPS-primed monocytes through the activation of the P2×7 receptor.^32^ In addition, LL-37 and its mouse homologue mCRAMP have been described to activate the NLRP3 inflammasome, in human and murine macrophages, respectively, leading to the release of mature IL-1β and IL-18.^16^ Both LL-37 and CRAMP have previously been purported to play an important role in the antiviral response to influenza infection,^33^ promoting viral clearance with an ensuing reduction in weight loss and death.

In our influenza model, both pro-form and cleaved form of mCRAMP were present in the BALF of influenza-treated mice 24 hours post infection and were strikingly reduced with neutrophil depletion. Furthermore, a significant correlation between the levels of BALF mCRAMP and BALF IL-1β supports the notion that neutrophil mCRAMP may drive IL-1β release from alveolar macrophages. Accordingly, mCRAMP stimulation of imiquimod-primed alveolar macrophages, but not neutrophils, resulted in IL-1β release. Collectively, these results show evidence for the neutrophil peptide mCRAMP activating the inflammasome in alveolar macrophages and promoting IL-1β release. While our data strongly support the notion that mCRAMP can drive alveolar macrophage IL-1β release during influenza infection, it is feasible that other neutrophil-derived signals may also contribute. Thus, in essence, a neutrophil-derived product can act as a danger signal to re-enforce macrophage activation and commit the macrophage to a potent effector pathway.

Neutrophil depletion also ablated BALF IL-1β at 24 hours post RSV infection, but no reduction in BALF IL-1β was seen in neutrophil-depleted mice treated with LPS, Hib or SP. Collectively, these data indicate that respiratory viral infection, but not bacterial infection, elicits a robust release of IL-1β that is critically dependent on neutrophil recruitment. Both influenza virus and RSV use the NLRP3 inflammasome to dictate IL-1β release.^20 21 42^ However, both LPS^43^ and SP^44^ also use the NLRP3 inflammasome, suggestive that this neutrophil-dependent IL-1β phenotype is not inflammasome complex specific. Instead, it seems probable that disparate infection models use distinct inflammasome activation signals or that there are additional redundant activation signals in bacterial models. Indeed, SP virulence factor pneumolysin is known to directly provide the second signal that activates the inflammasome,^18 45^ making unnecessary the need for signal provision from an exogenous source.

## Conclusion

We demonstrate that neutrophils recruited into the airways during viral infection release mCRAMP, which subsequently activates the NLRP3 inflammasome in alveolar macrophages and triggers IL-1β release. In the future, it will be important to assess the relevance of these studies to human disease and to perform confirmatory experiments using human cells and assess correlations among neutrophils, LL-37 and IL-1β in patients with respiratory viral infection. We conceptualise that the described pathway represents a novel mechanism by which neutrophils are recruited to confront a viable pathogenic threat and subsequently play a decisive role in liberating alveolar macrophages from their hyporesponsive state and amplifying inflammation. While this neutrophil-alveolar macrophage crosstalk may be critical to protective immunity, excessive neutrophil recruitment may result in overexuberant inflammasome activation and tissue pathology. Furthermore, it is feasible that this pathway may contribute to viral driven exacerbations of chronic lung diseases such as COPD and asthma.
