# Supplementary material for: Neutrophils drive alveolar macrophage IL-1β release during respiratory viral infection
Source: Thorax. 2017 Oct 27;73(6):546–56. doi: 10.1136/thoraxjnl-2017-210010 (PMC5969338; doi:10.1136/thoraxjnl-2017-210010)
Supplement: Supplementary file 1 [file thoraxjnl-2017-210010supp001.pdf]

Neutrophils drive alveolar macrophage IL-1β release during respiratory viral infection.

Teresa Peiró[1], Dhiren F. Patel[1], Samia Akthar[1], Lisa G. Gregory[1], Chloe J. Pyle[1], James A. Harker[1], Mark A. Birrell[2], Clare M. Lloyd[1], Robert J. Snelgrove[1,*].

Supplementary Material.

Supplementary Figures and Legends

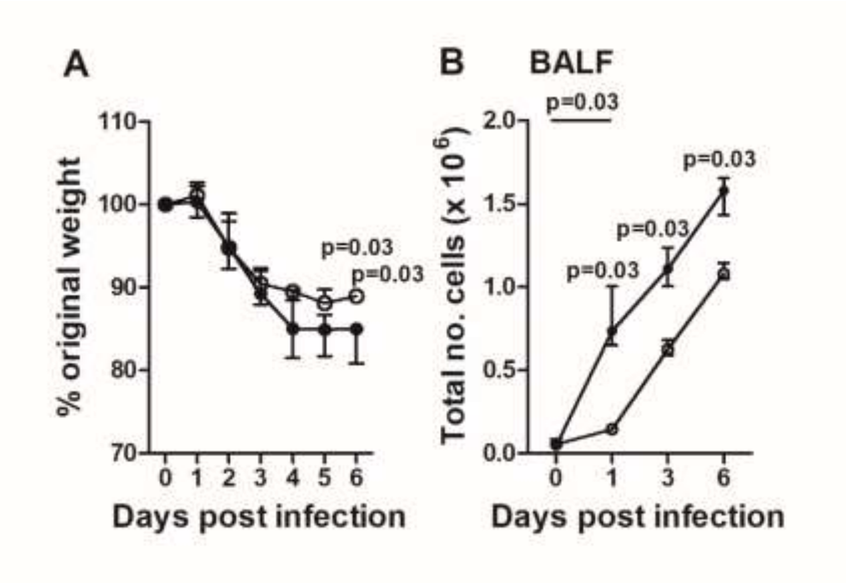

Supplementary Figure 1. Early neutrophils infiltrate contributes to influenza-driven illness and immunopathology. Mice were administered neutrophil-depleting (1A8) (open symbols) or control (2A3) antibody (filled symbols) intraperitoneally (i.p.) and inoculated with influenza X31 ($1x10^5$ pfu in 50 µl PBS) or PBS intranasally (i.n.) and culled at days 1, 3 and 6 post-infection. (A) Weight loss, depicted as percentage loss of original body weight, was determined each day post infection. (B) Total cells in BALF was assessed by Trypan blue exclusion. Data (median ± IQR) from 1 experiment with 4 mice per group and representative of 2 independent experiments. P values were calculated using the Mann-Whitney test.

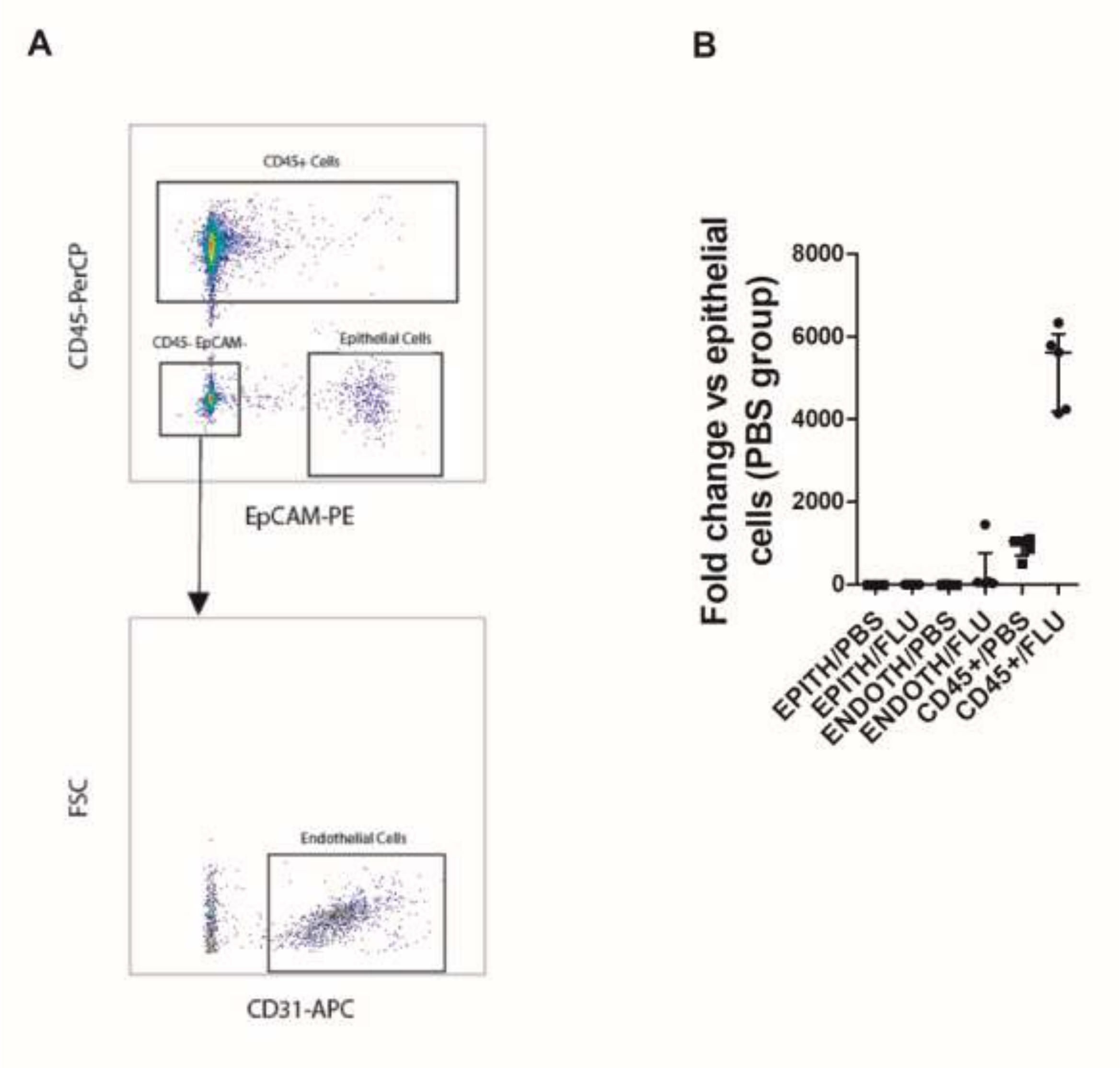

Supplementary Figure 2. Hematopoietic cells preferentially express IL-1β. Mice were administered influenza X31 (1x10^5 pfu in 50 µl PBS) or PBS intranasally (i.n.) and culled at 24 hrs post-infection. (A) Hematopoietic cells (CD45$^+$), epithelial cells (CD45$^-$ EpCAM$^+$) and endothelial cells (CD45$^-$ EpCAM$^-$ CD31$^+$) were isolated from digested lung tissue by fluorescent-activated cell sorting. (B) IL-1β mRNA within each population were assessed by real-time PCR and expressed as a fold increase relative to the epithelial PBS group. (B) Data (median ± IQR) from 1 experiment with 5 mice per group.

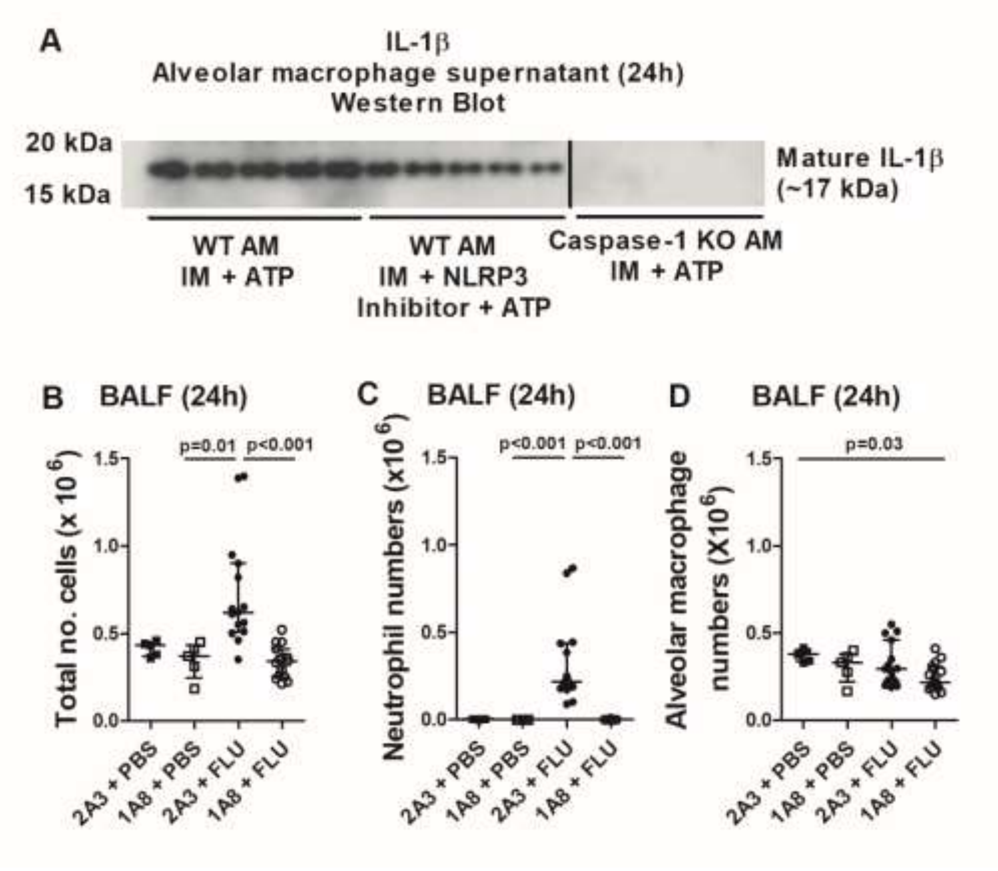

Supplementary Figure 3. Airway neutrophil and alveolar macrophages numbers are comparable 24 hours after influenza infection. Alveolar macrophages (AM) from wild-type (WT) or Caspase-1 knock-out (KO) mice were primed with imiquimod (IM) (1µg/ml) or IM + NLRP3 inhibitor (CP-456773; 50nM) for 4 hours followed by stimulation with ATP 1mM for 1 hour. (A) Release of mature IL-1β into supernatant was assessed by Western blot. The vertical black line depicted to indicate the grouping of images from different parts of the same gel. Mice were administered neutrophil-depleting (1A8) or control (2A3) antibody i.p. and inoculated with influenza X31 ($1x10^5$ pfu in 50 µl PBS) or PBS i.n. and culled 24 hours later. The numbers of (B) total cells, (C) neutrophils and (D) alveolar macrophages in the airways were determined by flow cytometry. (B-D) Data (median ± IQR) from 3 experiments with 5 mice per group. P values were calculated using Kruskal-Wallis with Dunn's post test.

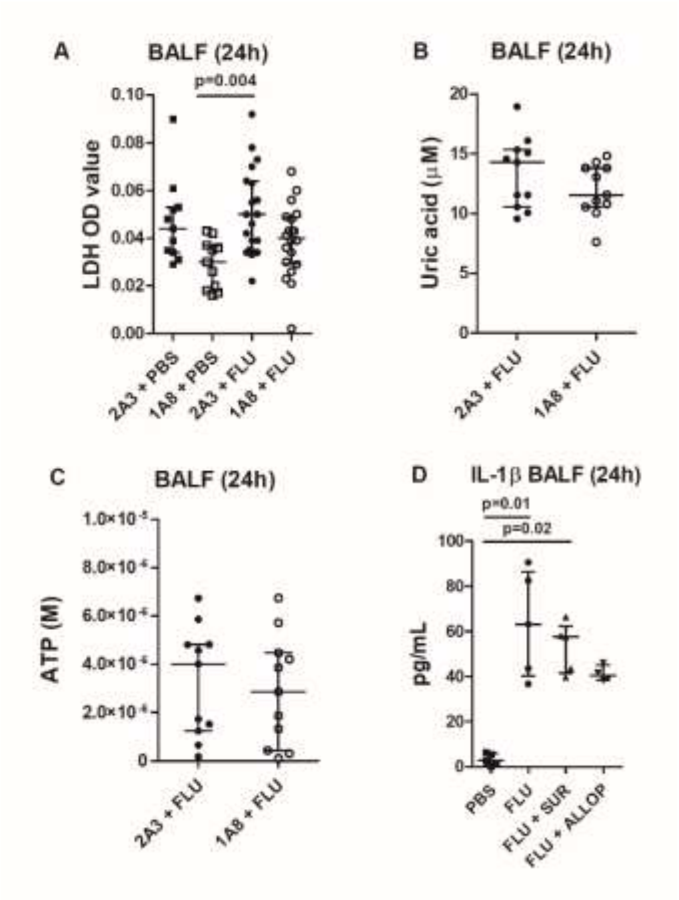

Supplementary Figure 4. Neutrophil-driven IL-1β release during influenza infection is independent of the generation of DAMPs. Mice were administered neutrophil-depleting (1A8) or control (2A3) antibody i.p. and inoculated with influenza X31 ($1x10^5$ pfu in 50 µl PBS) or PBS i.n. and culled 24 hours later. (A) LDH activity and the concentrations of (B) uric acid and (C) ATP in the BALF were determined. Mice were treated with suramin (SUR; 2mM in 50 µl PBS i.n.) or allopurinol (ALLOP; 25 mg/kg in 500 µL PBS i.p.) and inoculated with influenza X31 ($1x10^5$ pfu in 50 µl PBS) or PBS i.n. 1 hour after treatment. The ALLOP group received another dose 8 hours after infection. (D) Total IL-1β in the BALF was determined by ELISA. Figures represent (A) data combined of 4 experiments; (B,C) data combined of 2 experiments with at least 5 mice per group; (D) data from 1 experiment with 5 mice per group. Results depicted as median ± IQR. P values were calculated using Kruskal-Wallis with Dunn's post test.

Supplementary Methods

Pathogen stocks.

Influenza (strain X31), *Haemophilus influenzae b* (Hib) Eagan strain and *Streptococcus pneumoniae (S. pneumonia*; serotype 2) strain D39 (NCTC 7466, National Collection of Type Cultures, London, UK) were a kind gift from Professor T. Hussell (Institute of Inflammation and Repair, The University of Manchester, UK). Respiratory syncytial virus (RSV; strain A2) was obtained from the ATCC. Hib were cultured at 37°C in 5% CO2 in Brain Heart Infusion (BHI) broth (OXOID, Hampshire, UK) supplemented with 10 μg/ml of both Hemin (Roche, West Sussex, UK) and nicotinamide adenine dinucleotide (Sigma-Aldrich, Dorset, UK) or on BHI agar (OXOID) supplemented with 4% Levinthals. Levinthals was made by adding 50% horse blood (TCS Biosciences, Buckingham, UK) to BHI broth and heating to 70°C for 45 min. On cooling to 50°C, 0.7 mg/ml nicotinamide adenine dinucleotide was added and the supernatant was stored at -80 °C. Bacteria were cultured to an optical density $(OD)_{600}$ of 0.3 (~$1x10^9$ cfu/ml) and stored at -80°C in 10% glycerol as single-use aliquots. *Streptococcus pneumoniae* was cultured at 37°C in 5% CO2 on blood agar plates or in Todd-Hewitt broth (OXOID) supplemented with 0.5% yeast extract (OXOID) (THY broth) to an OD_{600} of 0.4 (~$1x10^8$ cfu/ml) and stored at -80°C in 10% glycerol as single-use aliquots.

Cell recovery and isolation.

Mice were administered 3 mg pentobarbital and exsanguinated via the femoral artery. The lungs were then inflated 5 times with 1.5 ml PBS via an intratracheal cannula and for bacteria experiments 100 μl from each mouse removed for enumeration of bacterial burden (see below). The remainder was centrifuged and the supernatant stored at –80°C. The pellet was resus-pended at 10^6 cells / ml in R10F (RPMI containing 10% (vol/vol) FCS, penicillin (50 U/ml), streptomycin (50 μg/ml) and L-glutamine (2 mM)) and cell viability was assessed using trypan

blue exclusion. Lung tissue was cut into small pieces and digested for 30 min at 37°C while agitated with 0.14 Wunsh unit of Liberase III enzyme (Roche, Florence, SC, USA) in the presence of 50 µg/ml of DNase (Roche) in RPMI containing 5% (vol/vol) FCS and disrupted to a single cell suspension by passage through a 100 µM sieve (BD labware, NJ, USA). The cell suspension was centrifuged for 5 min at 800xg and red blood cells lysed by resuspending pellets in ACK buffer (0.15M ammonium chloride, 1M potassium hydrogen carbonate and 0.01mM EDTA, pH 7.2) for 3 minutes at room temperature before spinning (800g for 5 min) and washing with R10F. Cell viability was assessed by trypan blue exclusion and cells resuspended in R10F at 10^6 cells/ml.

To maximize alveolar macrophages isolation for subsequent *in vitro* analysis (see below), the aforementioned lavage process was conducted with PBS containing 1mM EDTA and was repeated a further two times. Cells were subsequently combined from the three separate lavages and resuspended in R10F. The cell suspension was centrifuged (800xg, 5 min) and red blood cell lysed as described above. Cells were resuspended in R10F media and total cells were counted in a hemocytometer, assessing cell viability using trypan blue exclusion. When combining lavages from infected mice, alveolar macrophages were isolated and enriched by MACS separation (see below).

To isolate epithelial cells and endothelial cells, lungs were then inflated with 1.5 ml dispase II (5 mg/ml; Roche) and then were allowed to collapse naturally. Low-melting-point agarose (0.5 ml of 1% (wt/vol) agarose) was then slowly injected into the lungs and was immediately solidified by packing of the lungs in ice. Lungs were then removed and incubated for 40 min in dispase solution and were transferred to DMEM containing DNAse I (50 µg/ml; Roche), and the digested tissue was 'teased away' from the upper airways. Digested lung tissue was disrupted into single-cell suspensions by passage through a 100 µm sieve (BD Labware). Cell suspensions were stained with anti–mouse CD45-PerCP (eBioscience, Chesire, UK; 45-0451-

82; 1/100 dilution), anti-mouse EpCAM-PE (eBioscience, Chesire, UK; 12-5791-82; 1/100 dilution) and anti–mouse CD31-APC (eBioscience, Chesire, UK; 17-0311-82) as detailed below, and populations (endothelial cells: $CD45^-$ EpCAM $^-CD31^+$; epithelial cells: $CD45^-$ $CD31^-$ $EpCAM^+$; hematopoietic cells $CD45^+$ $CD31^-$ $EpCAM^-$) isolated by fluorescent-activated cell sorting (FACS) on a FACS Aria III. Cells were resuspended in RLT (Qiagen, Manchester, UK) for real time PCR (see below).

To isolate bone marrow neutrophils, the femur and the tibia from both hind legs were removed and freed of soft tissue attachments, and the extreme distal tip of each extremity cut off. HBSS containing 15mM EDTA and 30mM HEPES was forced through the bone with a syringe. After dispersing cell clumps and passage through a 100 µM sieve (BD labware), red blood cells were lysed by resuspending pellets in ACK buffer (for 3 minutes at room temperature) before centrifugation (800xg 5 min) and washing with HBSS. The cell suspension was centrifuged (800xg, 5 min, 4°C) and resuspended in RPMI. The cells were layered onto a 72%, 64%, 52% Percoll gradient (Sigma-Aldrich) diluted in PBS (100% Percoll = nine parts Percoll and one part 10x PBS), and centrifuged (1500xg, 30 min, room temperature) without braking. The neutrophils at the 64%/72% interface were harvested and washed with 20 ml RPMI. Cell viability was assessed by trypan blue exclusion and confirmed to be > 90% neutrophils by flow cytometry.

Influenza-specific plaque assay.

Lung homogenates were frozen and thawed three times and centrifuged at 4,000xg, and supernatants were ‘titrated’ in doubling dilutions on Madine-Darby canine kidney cell monolayers in flat bottomed 96-well plates. After incubation for 3 h at 37°C, samples were overlaid with 1% (wt/vol) methycellulose and were incubated for 72 h at 37°C. Cell monolayers

were washed and were incubated with anti-influenza (Serotec, Kidlington, UK) followed by horseradish peroxidase–conjugated anti-mouse (Dako, Cambridgeshire, UK), and infected cells were detected with 3-amino-9-ethylcarbazole substrate. Infectious units were counted by light microscopy and total plaque-forming units per lung were quantified (plaques x dilution factor x total volume of lung homogenate).

Bacterial cfu enumeration.

BALF and lung homogenate was serially diluted in sterile PBS and plated onto appropriate agar plates (Columbia blood agar plates for *S. pneumoniae*, BHI agar with 4% Levinthals for *Hib*) and grown overnight at 37°C with 5% CO_2. Resultant colonies were counted manually. *S. pneumoniae* was confirmed by optochin sensitivity. *Hib* was confirmed by no growth on BHI agar without Levinthals.

Flow cytometry.

Single-cell suspensions were stained for surface markers in PBS containing 0.1% sodium azide and 1% BSA for 30 min at 4 °C and fixed with 2% paraformaldehyde. Data were acquired on a BD FACS Fortessa machine (BD Bioscience, Oxford, UK). Forward-scatter and side-scatter gates were used to exclude debris, and dead cells were excluded using a fixable near-infrared dead cell stain kit for 633 or 635nm excitation.

Alveolar macrophages and neutrophils were characterized by their forward- and side-scatter profiles and by their phenotypes, as follows: Alveolar macrophages ($CD11b^{low-int}CD11c^{high}F4/80^{high}$) and neutrophils ($Ly6G^{high}CD11b^{high}CD11c^{low}F4/80^{low}$). The antibodies used were the following: CD11b-PerCP (eBioscience, Chesire, UK; 45-0112; 1/400 dilution),

CD11c-APC (BD Bioscience; 550261; 1/200 dilution), F4/80 (eBioscience; 12-4801; 1/50 dilution) and Ly6G-FITC (BD Bioscience; 551460; 1/100 dilution). Data was analyzed with FACSDiva software (BD Bioscience).

Immunofluorescence.

IL-1β expression was assessed in paraffin sections (4 μm) of lung tissue fixed in formalin. Sections were dewaxed in xylene (twice, 5 min each), rehydrated through 100%, 90%, 75% ethanol to water, 30 seconds each stage, and incubated in 1X PBS (twice, 5 min each). Sections were then incubated in preheated 10mM sodium citrate and microwaved (3 times, 5 min each), washed twice in PBS and blocked for 20 minutes at room temperature with 10% donkey serum in PBS. Following incubation, sections were washed in PBS and incubated overnight with 10μg/mL goat anti-IL-1β AF-401-NA (R&D Systems, Oxon, UK) or goat IgG control antibody I-5000 (Vector Laboratories, Burlingame, CA, USA) in 1% normal mouse serum (NMS)/PBS. Following incubation, sections were washed with 0.1% PBS/Tween (twice, 5 min) and PBS (once, 5 min) and were then incubated for 45 minutes with secondary antibody donkey anti-goat IgG Alexa Fluor 488 conjugate (Invitrogen, Paisley, UK; A-11055) diluted 1/250 in 1% NMS/PBS. Sections were washed with 0.1% PBS/Tween and PBS and were mounted with Prolong Gold antifade reagent with DAPI (Molecular Probes, Paisley, UK; P36935) for fluorescence microscopy. Fluorescence images were acquired with a Leica microscope and Leica DFC 300 FX camera using the Leica Application Suite V4 software (Leica, UK).

MACS sorting.

BAL alveolar macrophages were sorted using MS MACS Columns and MACS MicroBeads (Miltenyi Biotec, Surrey, UK) according to manufacturer's instructions. Briefly, total cells obtained from the BAL were centrifuged (200xg for 10 minutes) and resuspended in MACS

buffer (PBS, pH=7.2, 0.5% BSA and 2 mM EDTA) containing CD11c MicroBeads (1/5 dilution) and incubated for 15 minutes at 2-8°C. Cells were subsequently washed, and resuspended in MACS buffer before being applied to pre-rinsed MS columns positioned in the magnetic field of a MACS Separator. For isolation of CD11c$^+$ macrophages, columns were removed from the magnetic field and cells flushed out with MACS buffer. CD11c+ cells (>85% purity) were resuspended in RLT (Qiagen, Manchester, UK) for real time PCR (see below).

Alveolar macrophage and bone marrow neutrophil *in vitro* stimulations.

Cells were seeded at 1 x10^5 per well and alveolar macrophages further enriched by adherence for 90 min in RPMI at 37°C, 5% CO_2. Cells were washed three times with R10F-PS (RPMI containing 10% (vol/vol) FCS, penicillin (50 U/ml), streptomycin (50 μg/ml) and L-glutamine (2 mM)). Cells were primed with 1 μg/mL TLR7 agonist Imiquimod R837 (Invivogen, Toulouse, France; tlrl-imqs), Imiquimod and 50 nM NLRP3 inhibitor CP-456773 (Sigma-Aldrich) or vehicle for 4 hrs (to mimic signal 1 required for inflammasome induction). Cells were subsequently washed with R10F-PS and cultured for an additional hour in new medium containing 1mM ATP (Sigma-Aldrich) or vehicle (to mimic signal 2 required for inflammasome activation). In some experiments, cells were seeded at 5 x10^5 per well, primed with Imiquimod for 4 hrs (as detailed above) and then stimulated for two additional hours in new medium with 20 μM mCRAMP (Innovagen, Lund, Sweden; SP-CRPS-1) or vehicle. Cell-free supernatants were collected for mediator analysis and western blot.

Mediator analysis.

The levels of cytokines (IL-1β, TNF-α, IL-6) in BALF, lung homogenates and cell-free culture supernatants were measured by enzyme-linked immunosorbent assay (eBioscience), following the manufacturer's instructions. The IL-1β ELISA utilized in these studies detects both pro-

and mature forms of the cytokine. LDH activity in BALF was assessed using the Lactate Dehydrogenase Activity Assay Kit (Sigma-Aldrich). Uric acid in BALF was measured using an Amplex red uric acid/uricase assay kit (Invitrogen). ATP in BALF was measured using an ATP Lite Detection Kit (PerkinElmer, Groningen, Netherlands).

Real-time PCR.

Total RNA was extracted from lung tissue (azygous lobe) or isolated cells using a Qiagen RNeasy Mini Kit or Qiagen RNeasy Micro Kit respectively. Total RNA was reverse transcribed into cDNA using a High Capacity cDNA Reverse Transcription Kit (Life Technologies, Paisley, UK) or GoScript™ Reverse Transcription System (Promega, Southampton, UK) as per the manufacturer's instructions. Real-time PCR reactions were performed using fast-qPCR mastermix (Life Technologies) on a Viaa-7 instrument (Life Technologies) with TaqMan primer sets for murine IL-1β, NLRP3, Caspase 1, ASC, HPRT, GAPDH (Life Technologies) and gene expression was analyzed using the change-in-threshold ΔΔCt method. Fold-changes in mRNA expression for targeted genes were calculated relative to control cells treated with vehicle or control mice administered with 2A3 antibody and treated with PBS.

Quantification of viral RNA.

Total RNA was extracted from the lung by use of RNA stat-60 (AMS Biotech Ltd.), and cDNA was generated with random hexamers by use of Omniscript reverse transcriptase (QIAGEN). Real-time PCR was carried out for the RSV L gene by use of 900 nM forward primer (5′-GAACTCAGTGTAGGTAGAATGTTTGCA-3′), 300 nM reverse primer (5′-TTCAGC-

TATCATTTTCTCTGCCAAT-3′), and 100 nM probe (5′-6-carboxyfluorescein-TTTGAAC-CTGTCTGAACAT-6-carboxytetramethylrhodamine-3′) on a Viaa-7 instrument (Life Technologies).

Western blot.

For detection of IL-1β by western blot, BALF (500μl; pooled 100 μl from each mouse) was precipitated with one volume of methanol and one-fourth volume of chloroform. Samples were centrifuged (5min; 15.000xg), the upper phase removed and the remaining lower phase washed with one volume of methanol and centrifuged again. The supernatant was removed and the pellet dried for 5 min at 50°C before being resuspended in LDS loading buffer (Novex, Life Technologies) with 10% mercaptoethanol (Sigma-Aldrich). For IL-1β immunoblot in cultured supernatants and for mCRAMP immunoblot in BALF, samples were directly combined with LDS loading buffer with 10% mercaptoethanol. All samples were subsequently incubated at 95° for 5 min, separated on 4–12% Criterion™ XT Bis-Tris precast polyacrylamide gels (BIORAD, Hertfordshire, UK) and proteins were transferred onto polyvinylidene difluoride membranes (ThermoFisher Scientific, Paisley, UK). Membranes were blocked (5% milk in Tris-buffered saline solution) for 1 hour at room temperature and were incubated overnight at 4°C with primary antibodies diluted in 5% milk in TRIS-buffered saline solution: polyclonal goat anti-IL-1β (1/800; R&D Systems, AF-401-NA) or polyclonal rabbit anti-mCRAMP (1/1000; Innovagen, PA-CRPL). Membranes were subsequently washed and incubated with donkey anti-goat IgG-horseradish peroxidase (HRP) (1/2500; Santa Cruz, sc-2020) or goat anti-rabbit IgG-HRP (1/2000; Cell Signaling, Danvers, MA, USA; 7074) diluted in 5% milk in TRIS-buffered saline solution. Reactivity was visualized by using the ECL2 Western Blotting Substrate kit (ThermoFisher Scientific), following manufacturer’s instructions.

Cytoplasmic fractions from lung homogenates were obtained using the CelLyic NuCLEAR Extraction Kit (N-XTRACT) following the manufacturer’s instructions (Sigma-Aldrich). Total

protein was determined by Bradford Protein Assay (BIORAD) and 2µg of total protein was loaded into 4–12% Criterion™ XT Bis-Tris precast polyacrylamide gels (BIORAD, UK). Electrophoresis, transfer and developing were performed as described above. Membranes were probed with primary antibodies anti-Caspase-1 (p20) Casper-1 (1/1000; Adipogen, Liestal, Switzerland; AG-20B-0042) or anti-alpha Tubulin antibody [EPR13478(B)] – loading control (1/1000; Abcam, Cambridge, UK; ab176560). Membranes were probed with secondary antibodies anti-mouse IgG HRP-linked (1/1000; Cell Signaling, 7076) or anti-rabbit IgG HRP-linked (1/2000; Cell Signaling, 7074).
